# Molecular Diagnosis and Identification of Equine Piroplasms: Challenges and Insights from a Study in Northern Italy

**DOI:** 10.3390/ani15030437

**Published:** 2025-02-05

**Authors:** Veronica Facile, Martina Magliocca, Filippo Maria Dini, Ilaria Imposimato, Jole Mariella, Francesca Freccero, Lorenza Urbani, Riccardo Rinnovati, Emily Sel, Laura Gallina, Carolina Castagnetti, Roberta Galuppi, Mara Battilani, Andrea Balboni

**Affiliations:** Department of Veterinary Medical Sciences, Alma Mater Studiorum-University of Bologna, Via Tolara di Sopra 50, 40064 Ozzano dell’Emilia, Bologna, Italy; veronica.facile2@unibo.it (V.F.); martina.magliocca2@unibo.it (M.M.); filippomaria.dini@unibo.it (F.M.D.); ilaria.imposimato2@unibo.it (I.I.); jole.mariella2@unibo.it (J.M.); francesca.freccero2@unibo.it (F.F.); lorenza.urbani2@unibo.it (L.U.); riccardo.rinnovati2@unibo.it (R.R.); emily.sel@studio.unibo.it (E.S.); laura.gallina@unibo.it (L.G.); carolina.castagnetti2@unbo.it (C.C.); roberta.galuppi@unibo.it (R.G.); mara.battilani@unibo.it (M.B.)

**Keywords:** *Babesia* spp., *Theileria* spp., horse, molecular diagnosis, 18S rRNA, *ema1*

## Abstract

Equine piroplasmosis is a tick-borne disease caused by *Babesia* and *Theileria* species. Despite its presence in Europe, no laboratory testing is required for animal movement, even though some countries remain free of this disease. Differentiating between species and genotypes is crucial to determine the most effective treatment, as dosage, active compounds, and duration vary. However, diagnosis is often challenging due to genetic variability and the limited sensitivity of molecular methods. The aims of this study were to compare the performances of different molecular diagnostic tests to identify the most effective assay for piroplasm DNA detection and to genetically characterize the piroplasms identified in horses in Northern Italy from 2016 to 2022. Among 63 horses tested, 24 (38.1%) were positive in at least one of the tests used. Four horses tested positive for *Babesia caballi* with identical nucleotide sequences, while 22 horses tested positive for genetically different *Theileria* species, including *Theileria equi*, *Theileria haneyi*-like species, and *Theileria* sp. *Africa*. Two horses were coinfected by *Babesia caballi* and *Theileria haneyi*-like species. The best diagnostic approach to avoid false negative results was a combination of different assays. Further studies will be necessary to better assess the prevalence and genetic diversity of piroplasms involved in equine piroplasmosis.

## Simple Summary

Equine piroplasmosis is a tick-borne disease with significant health and economic impacts on the equine industry. Various piroplasm species and genotypes belonging to *Babesia* and *Theileria* genera have been identified as causative agents of this disease in horses, and recent studies highlight treatment differences depending on the species involved. Therefore, knowing which piroplasm species are circulating in a specific area is crucial, and highly sensitive diagnostic methods are needed to identify and differentiate the pathogen responsible for the infection. The aims of our study were to compare the diagnostic performance of different molecular tests for piroplasm DNA detection and genetically characterize the piroplasms identified in 63 horses in Northern Italy from 2016 to 2022. Molecular analysis revealed a 38.1% positivity rate within the tested population. Notably, substantial genetic variability was observed among the identified theileria rather than among the babesia. No single diagnostic method was found to reliably detect and differentiate all piroplasm species involved in equine piroplasmosis. This study highlights the need for further investigation into the genetic diversity of these parasites. Expanding our understanding of piroplasm variability is essential to develop and implement appropriate diagnostic methods for the accurate detection of equine piroplasmosis.

## 1. Introduction

Equine piroplasmosis (EP) is a disease caused by tick-borne pathogens (TBPs) *Babesia caballi*, *Theileria equi,* and the recently discovered *Theileria haneyi* [1,2]. EP has a global distribution and is endemic in tropical and subtropical areas [3,4,5]. Few countries, such as Canada, the United States, the United Kingdom, Japan, and Australia, remain EP-free or have reported only sporadic cases [6]. In Italy, a molecular prevalence between 1% and 5% for *B. caballi* and 35% for *T. equi* was reported [5]. These data highlight the importance of testing equids before international movement to prevent sanitary consequences and economic repercussions in the horse industry [7]. As already reported by Nardini and colleagues [8], the European Union legislation states that only a clinical evaluation of the absence of symptoms of equine piroplasmosis is required as proof of freedom from infection, and no laboratory confirmation is required for animal movements (Council Directive 2009/156/EC). While *Babesia* species replicate asexually within red blood cells and are capable of transovarial transmission in ticks, *Theileria* species exhibit a pre-erythrocytic stage in leukocytes and rely exclusively on transstadial transmission [9]. Given their pathogenesis, infections frequently manifest as hematological abnormalities. In cases of *Babesia* infections, these are typically acute forms presenting with more severe clinical signs, including pyrexia, malaise, dehydration, congested mucous membranes, petechiae, ecchymoses, limb edema, and anemia. Conversely, *Theileria* infections are usually chronic in nature, characterized by milder and less specific clinical signs such as anorexia, weight loss, general malaise, and mild anemia [10]. The diagnosis of EP is often challenging, as various techniques are employed to identify the piroplasms. Blood smear microscopy is a simple and easy-to-perform method that allows direct identification of parasites within the erythrocytes of the infected horse. However, interpretation can be subjective, requiring specialized personnel. Additionally, this method has low sensitivity, even during the acute phases of the disease, due to the often low parasitemia observed in EP cases [10]. On the other hand, serological methods (complement fixation test—CFT, enzyme-linked immunosorbent assay—ELISA, immunochromatographic test—ICT, Western blot—WB, and indirect immunofluorescence assay test—IFAT) are the most commonly used, especially in large-scale studies [4]. These methods assess the antibody titers in infected subjects but may yield false-negative results during the acute stages [11,12]. Finally, molecular techniques are highly sensitive from the early stages of infection, and they can detect small quantities of the parasite DNA in carrier hosts and in the chronic phases of infection [9]. Furthermore, these assays allow for genetic characterization of the pathogen involved in the infection through phylogenetic analyses [13]. Given the high genetic diversity, the classification of these piroplasms is often challenging, and furthermore, to date, no single sensitive and specific assay has been reported able to identify and differentiate the various species of parasites responsible for equine piroplasmosis [14,15]. The 18S ribosomal RNA (18S rRNA) is the most commonly used gene for the taxonomic classification of these parasites, but it is often insufficient for accurate species identification, especially concerning *Theileria* spp. [16]. In fact, for this piroplasm, the equine merozoite antigen 1 (*ema1*) gene, coding for one of the most important surface antigens in *T. equi*, is frequently used as an additional target [17]. Nevertheless, Knowles and colleagues [2] reported that *T. haneyi* lacks this specific gene, meaning that analyses targeting it may fail to identify this newly recognized species. The aims of this study were to compare the performances of different molecular diagnostic tests for equine piroplasmosis to identify the most effective assay and to genetically characterize the *Babesia* and *Theileria* species identified in horses in Northern Italy from 2016 to 2022.

## 2. Materials and Methods

### 2.1. Study Population

Horses exposed to risk factors (environment, breed, presence of ticks) for piroplasms infection or with clinical suspicion of EP referred and tested for piroplasm DNA detection at the Veterinary Teaching Hospital (VTH) of the University of Bologna (Ozzano Emilia, Bologna, Italy) (latitude 44.43612143900343, longitude 11.486610096950223), between 2016 and 2022, were retrospectively included in the study. All the included horses originated from Northern Italy; continental climatic zones were characterized by temperate climates. Over the years, this area has experienced various climatic changes that have influenced the local ecology, increasing the presence of vectors and, consequently, the diseases they transmit [18]. The definition for a clinical suspicion was correlated to the presence of at least one of the following clinical signs: depression, poor performance, fever, jaundice, inappetence, and edema [9], associated with low erythrocyte counts, low hemoglobin, low platelets, or high bilirubin [19]. Year of sampling and signalment data (sex and age) of the enrolled horses were retrieved from medical records. Horses included were grouped according to three different age categories: (i) newborn foal (≤7 days); (ii) colt/filly (8 days–12 months); (iii) adult horses (≥12 months).

### 2.2. Molecular Detection

DNA was extracted from blood samples using the NucleoSpin Tissue Kit (Macherey-Nagel, Düren, Germany), according to the manufacturer’s instructions, at the time of referring to VTH for piroplasms DNA detection. The extracted DNA was stored at −20 °C until use for subsequent analysis. DNA extracted from blood samples of all the horses included in the study were tested by three different molecular assays for *Babesia* spp. or *Theileria* spp. DNA detection. Sensitivity and specificity of nested PCR, multiplex PCR, and real-time PCR were evaluated and compared to each other. The different molecular assays used are summarized in Table 1.

#### 2.2.1. Nested PCR

A nested PCR (nPCR) designed on the 18S rRNA gene and previously developed by Jefferies and colleagues [20] was used to detect *Babesia* and *Theileria* species amplifying a unique 800 base pair (bp) DNA fragment without distinction between the two piroplasms genera. The reactions were carried out using the Taq DNA Polymerase Kit (Qiagen, Hilden, Germany), according to the manufacturer’s instructions. The thermal cycling of the first PCR reaction consisted of an initial denaturation at 94 °C for 5 min followed by 45 cycles of denaturation at 94 °C for 1 min, annealing at 58 °C for 1 min, and elongation at 72 °C for 1 min and 10 s, followed by a final elongation step at 72 °C for 10 min. A second PCR reaction was performed using PCR products of the first amplifications with the following thermal cycle: initial denaturation at 94 °C for 5 min followed by 45 cycles of denaturation at 94 °C for 1 min, annealing at 62 °C for 1 min and elongation at 72 °C for 1 min, followed by a final elongation step at 72 °C for 10 min. Two samples that tested positive for *Babesia* and *Theileria* species DNA, respectively, were used as internal positive control, and a no template control, consisting of ultrapure water, underwent analysis simultaneously. PCR products were visualized under UV after electrophoresis migration on a 1.5% agarose gel stained with Midori Green Advance DNA Stain (Nippon Genetics, Düren, Germany) in 1× standard tris-acetate-EDTA (TAE) buffer. Amplicons of the expected size were considered positive.

#### 2.2.2. Multiplex PCR

A multiplex PCR (mPCR) designed on the 18S rRNA gene and previously developed by Alhassan and colleagues [21] was used to detect and distinguish *B. caballi* and *T. equi* on the basis of the amplicon size, producing a 540 bp DNA fragment for *B. caballi* and a 450 bp DNA fragment for *T. equi*. The reactions were carried out using the Taq DNA Polymerase Kit (Qiagen, Hilden, Germany), according to the manufacturer’s instructions. The thermal cycling consisted of an initial denaturation at 94 °C for 5 min, followed by 45 cycles of denaturation at 94 °C for 1 min, annealing at 60.5 °C for 1 min, and elongation at 72 °C for 1 min, and a final elongation step at 72 °C for 10 min. An internal positive control and a no template control, consisting of ultrapure water, underwent analysis simultaneously. PCR products were visualized under UV after electrophoresis migration on a 1.5% agarose gel stained with Midori Green Advance DNA Stain (Nippon Genetics, Düren, Germany) in 1× standard tris-acetate-EDTA (TAE) buffer. Amplicons of the expected size were considered positive.

#### 2.2.3. Real-Time PCR

An SYBR Green real-time PCR (qPCR) designed on the large subunit ribosomal DNA (*lsu*) and developed by Qurollo and colleagues [22] was used to detect all the *Babesia* species by amplifying a 150 bp DNA fragment for *Babesia* spp. and no product for *Theileria* spp. The reactions were carried out using the PowerUp SYBR Green master mix (Thermo Fisher Scientific, Waltham, MA, USA) in a total volume of 20 μL and the Real Time StepOnePlus™ system (Applied Biosystem, Carlsbad, CA, USA). The thermal cycling consisted of DNA polymerase activation at 95 °C for 5 min and 45 cycles at 95 °C for 15 s and 60 °C for 1 min. Melting experiment for the evaluation of the specificity of the reaction was performed after the last extension step by a continuous increment from 60 °C to 95 °C, and specific melting temperature (Tm) was about 73 °C. DNA copy number determination was carried out by absolute quantification using the standard curve method. Serial 10-fold dilutions of a plasmid (pCR4 plasmid; Thermo Fisher Scientific, Life Technologies, Carlsbad, CA, USA) containing one copy of the target sequence were used as external standards for the construction of the assay standard curve by plotting the plasmid copy number against the corresponding threshold cycle values. The limit of detection (LOD) of the reactions was determined based on the highest dilution of recombinant plasmid possible to amplify with good reproducibility and was found to be 1 DNA copy/µL. The DNA samples and standards were repeated within each run in duplicate. A no-template control, consisting of ultrapure water, underwent analysis simultaneously. Samples showing an exponential increase in the fluorescence curve, a target DNA amount greater than or equal to the LOD, and a specific melting peak in both replicates were considered positive.

### 2.3. Genetic Characterization

According to the method proposed by Knowles and colleagues [2] to characterize and distinguish *T. equi* and *T. haneyi*, two other end-point PCR assays were carried out on the DNA extracts tested positive for *Theileria* spp. DNA. The first PCR targeted a 229 bp DNA fragment of the *ema1* gene that is reported specific exclusively for *T. equi* and not for *T. haneyi* [23,24], and the second one targeted a 238 bp DNA fragment of a 2118 bp single-copy ORF of chromosome 1 that is reported exclusively for *T. haneyi* and not for *T. equi* [2,24,25] (Table 1). The reactions were carried out using the Taq DNA Polymerase Kit (Qiagen, Hilden, Germany), according to the manufacturer’s instructions. Both thermal cycling consisted of an initial denaturation at 94 °C for 5 min followed by 40 cycles of denaturation at 94 °C for 1 min, annealing at 54 °C for 30 s and elongation at 72 °C for 30 s, followed by a final elongation step at 72 °C for 7 min. An internal positive control and a no template control, consisting of ultrapure water, underwent analysis simultaneously. PCR products were visualized under UV after electrophoresis migration on a 2% agarose gel stained with Midori Green Advance DNA Stain (Nippon Genetics, Düren, Germany) in 1× standard tris-acetate-EDTA (TAE) buffer. Amplicons of the expected size were considered positive.

Amplicons of the expected size obtained with all the qualitative PCR assays carried out in this study were purified using the QIAquick PCR Purification Kit (Qiagen, Hilden, Germany) according to the manufacturer’s instructions and directly sequenced by Sanger method (BioFab Research, Rome, Italy) using both forward and reverse primers. The nucleotide sequences obtained were assembled and translated into amino acid sequences using BioEdit sequence alignment editor version 7.2.5. The assembled nucleotide sequences were analyzed using the BLAST web interface (https://blast.ncbi.nlm.nih.gov/Blast.cgi (accessed on 15 November 2024)) to determine which species they belonged to and aligned with reference sequences available in the GenBank database (https://www.ncbi.nlm.nih.gov/nucleotide/ (accessed on 15 November 2024)), using the ClustalW method implemented in the BioEdit software alignment editor version 7.2.5.

Phylogeny was carried out with the MEGA 11 software version 11.0.11 [26] using the Neighbor-Joining method and the Kimura 2-parameter model for both the 18S rRNA gene of *Babesia* and *Theileria* species and the *ema1* gene of *Theileria* spp. The robustness of individual nodes on the phylogenetic trees was estimated using 1000 bootstrap replicates, and relevant bootstrap values were indicated at the corresponding nodes.

### 2.4. Statistical Analysis

All the retrieved data were captured in Microsoft Excel 2019 and analyzed using commercially available statistical software (MedCalc Statistical Software version 19.5.1, Ostend, Belgium). Descriptive statistics was performed for all the evaluated variables, and data are reported as mean ± standard deviation or median and range (minimum–maximum values), based on their distribution. Categorical data, such as year of sampling, sex, and age groups, were analyzed using Fisher’s exact *p*-value test or Pearson’s chi-squared (χ2) test. Continuous data (e.g., age) were analyzed using the Mann–Whitney test.

In the absence of a gold standard, a comparison between the diagnostic molecular assays was performed by estimating their relative sensitivity (rSe) and relative specificity (rSp), considering those animals that tested positive in at least one molecular test as infected and those animals that tested negative as non-infected in all molecular tests used. The rSe and rSp of the three assays for *Babesia* spp. and *Theileria* spp. detection were estimated as follows:

(1) $$\mathrm{rSe}:\;\frac{{Positive}_{A}}{{Positive}_{TOT}}\times 100,$$

(2) $$\mathrm{rSp}:\;\frac{{Negative}_{A}}{{Negative}_{TOT}}\times 100,$$

rSe is estimated by the ratio in percentage between the number of positive test outcomes of an assay (A) for *Babesia* spp. or *Theileria* spp. and the number of positive test outcomes in at least one molecular test. rSp is estimated by the ratio in percentage between the number of negative test outcomes of an assay (A) for *Babesia* spp. or *Theileria* spp. and the number of negative test outcomes in at least one molecular test. The 95% confidence interval was also calculated for the sensitivity and specificity values using the following formula [27]:

(3) $$\mathrm{IC}95\%:\pm 1.96\sqrt{\begin{array}{c} \frac{Se\left(1-Se\right)}{n} \end{array}}\;\;\;\;\;\;\pm 1.96\sqrt{\begin{array}{c} \frac{Sp\left(1-Sp\right)}{n} \end{array}},$$

## 3. Results

### 3.1. Study Population

**Figure 1:**
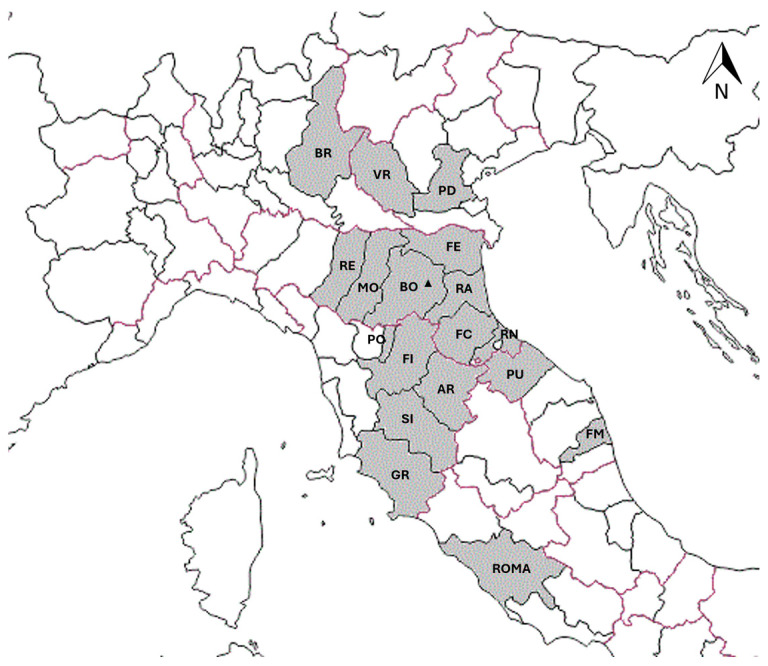
Italian provinces of origin of the horses included in the study. Outlined in purple = Italian regions; outlined in black = Italian provinces; highlighted in grey = Italian provinces of origin of the horses included in the study. AR = Arezzo; BO = Bologna; BR = Brescia; FC = Forlì-Cesena; FE = Ferrara; FI = Firenze; FM = Fermo; GR = Grosseto; MO = Modena; PD = Padova; PO = Prato; PU = Pesaro–Urbino; RA = Ravenna; RE = Reggio Emilia; RN = Rimini; ROMA = Roma; SI = Siena; VR = Verona. ▲ = location of the VTH.

During the study period, 63 horses were retrospectively included. The total number of horses suspected of infection progressively increased over the years. In the first two years of the study (2016–2017), 4 out of 63 horses (6.3%) were tested, while a significant increase was observed by the last year of the study (2022), with 21 (33.3%) horses tested (*p* < 0.001). Among the horses included, 30/63 (47.6%) were male (10/30, 33.3% gelding) and 33/63 (52.4%) were female (none spayed). The median age was 9 years and 5 months (range 1 month—28 years). The horses included in the study originated from various provinces in Northern Italy (Figure 1). No correlation was observed between the area of origin and positivity to the different piroplasm species.

### 3.2. Molecular Detection

Twenty-four out of the sixty-three horses (38.1%) tested positive in at least one of the three assays used for molecular detection of piroplasms DNA. A significant association was found between positive results to at least one pathogen investigated and the sex of horses, with females (17/33, 51.5%) more frequently being positive than males (7/30, 23.3%) (*p* = 0.0368). The median age of horses that tested positive was 9 years and 6 months (range 1 month—28 years) with no significant difference between age categories.

From molecular assay and sequencing results, 4/24 positive horses were infected by *Babesia* spp., and 22/24 were infected by *Theileria* spp. Two of these positive horses were coinfected by the two pathogens. The complete results obtained through molecular methods and sequencing are summarized in Table 2.

In Table 3, sensitivity and specificity values, along with their respective confidence intervals, were reported for each molecular assay used for *Babesia* spp. and *Theileria* spp. DNA detection.

### 3.3. Genetic Characterization

Sequence analysis of the 18S rRNA gene, on a fragment of about 580 bp for *Babesia* spp. and 680 bp for *Theileria* spp., revealed that the four horses that tested positive for *Babesia* spp. were infected by *B. caballi* (4/63, 6.4%) and the sequences obtained were identical to each other. On the other hand, the 22 horses that tested positive for *Theileria* spp. were infected by piroplasms belonging to three distinct genetic groups: (i) nine horses (9/63, 14.3%) were infected with *T. equi*; (ii) six horses (6/63, 9.5%) were infected with piroplasms displaying nucleotide profiles compatible with some *T. haneyi* previously reported in China (GenBank ID: ON429000, ON429018, ON429006) and some *T. equi* (GenBank ID: AY534882, DQ287951), hereinafter referred to as *T. haneyi*-like; and (iii) seven horses (7/63, 11.1%) were infected with piroplasms that exhibited numerous unique and shared nucleotide mutations, showing significant similarities to sequences deposited in GenBank as *Theileria* sp. *Africa*, a novel candidate species that has not yet been classified. Two horses (2/63, 3.2%) were co-infected with *B. caballi* and *T. haneyi*-like.

**Figure 2:**
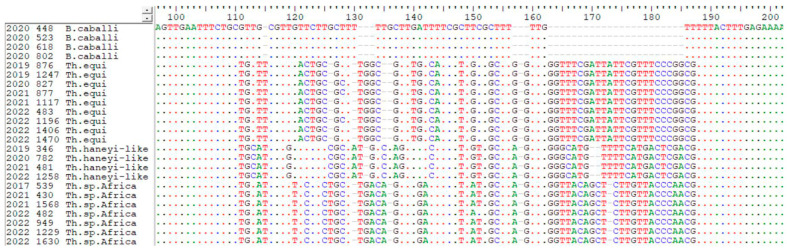
Nucleotide alignment of a fragment of the V4 hypervariable region of the18S rRNA gene of the sequences obtained from the piroplasms identified in this study.

Sequencing results are presented in Table 4, and Figure 2 shows a portion of the nucleotide alignment of the 18S rRNA gene constructed with sequences obtained in this study.

As reported by Nehra and colleagues [15], the fragment between nucleotides 113 and 183 of the V4 hypervariable region of the 18S rRNA gene allows for the differentiation of species and the genotyping of piroplasms.

Moreover, 16 out of 22 (72.7%) *Theileria*-positive samples tested positive with the *T. equi*-specific PCR assay targeting a fragment of the *ema1* gene. Specifically, all the nine *Theileria* identified as *T. equi* on the basis of 18S rRNA gene analysis tested positive, as 2/6 *T. haneyi-like* samples and 5/7 *Theileria* sp. *Africa*. Contrariwise, none of the *Theileria*-positive samples tested positive with the *T. haneyi*-specific PCR assay targeting a fragment of the ORF of chromosome 1.

**Figure 3:**
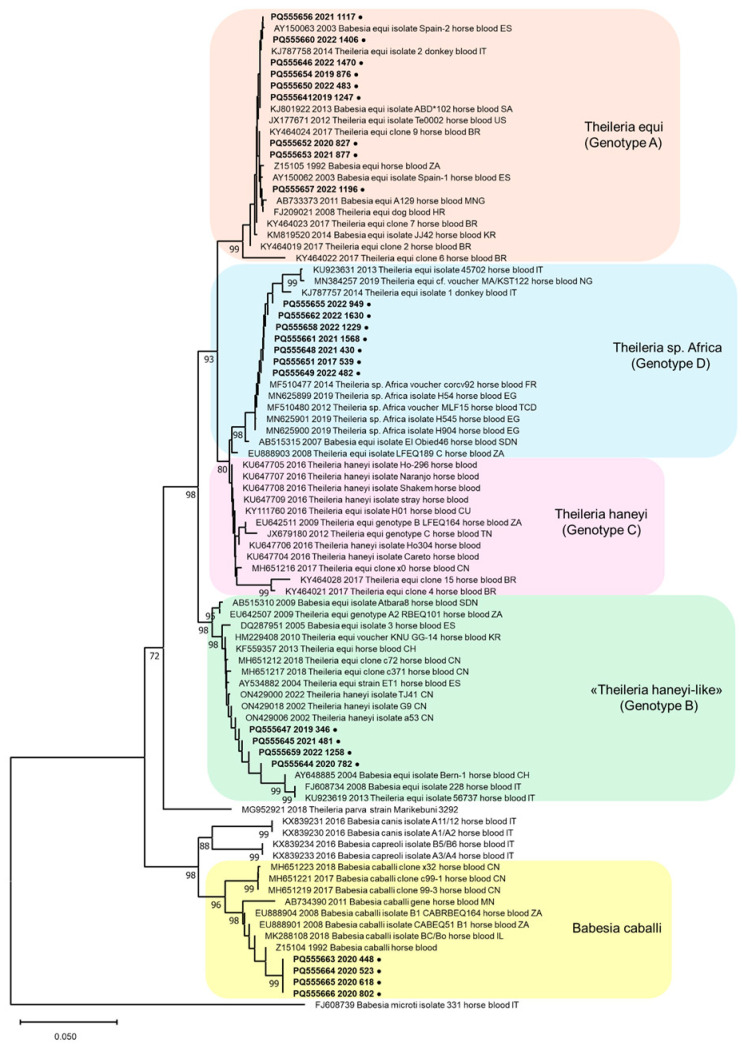
Phylogenetic tree constructed using the Neighbor-Joining method and the Kimura 2-parameter model implemented in MEGA 11 software version 11.0.11, based on the nucleotide alignment of 727 residues of the 18S rRNA gene of *Babesia* spp. and *Theileria* spp. sequences obtained in this study by sequencing the nested PCR products, with 14 reference sequences of *Babesia* spp. and 49 reference sequences of *Theileria* spp. available in GenBank (https://www.ncbi.nlm.nih.gov/nucleotide/ (accessed on 15 November 2024)). Bootstrap values calculated from 1000 replicates are indicated on the respective branches. The reference sequences are labeled as follows: GenBank number, date of collection (or submission date), genus, species, host, sample matrix, country of origin (BR = Brazil, CH = Switzerland, CN = China, CU = Cuba, ES = Spain, EG = Egypt, FR = France, HR = Croatia, IL = Israel, IT = Italy, KR = South Korea, MN = MNG = Mongolia, NG = Nigeria, SA = Saudi Arabia, SDN = Sudan, TCD = Chad, TN = Tunisia, US = United States, ZA = South Africa) ● = sequences obtained in this study labeled with GenBank number (PQ555641-PQ555666), year of collection and sample ID. The main species of piroplasms and (the corresponding genotype proposed for *Theileria* genus by Nehra and colleagues [15]) characterizing the clusters are reported alongside the tree.

The phylogenetic tree constructed with a nucleotide alignment of 727 residues of the 18S rRNA gene of *Babesia* and *Theileria* species (Figure 3) revealed that the *B. caballi* identified in this study clustered together with other *B. caballi* sequences in a distinct group separated from *B. capreoli* and *B. canis* sequences. Regarding the sequences of *Theileria* spp., phylogeny confirmed the presence of four distinct groups, as reported by Nehra and colleagues [15]. The strains identified in this study clustered in three of these different groups (Figure 3). Nine *Theileria* identified in this study clustered with *T. equi* reference sequences, with a nucleotide identity ranging from 96.6% to 100%. Four *Theileria* clustered with some *T. haneyi*-like reference sequences, with a nucleotide identity ranging from 98.9% to 100%. Seven *Theileria* clustered with *Theileria* sp. Africa reference sequences identified in Egypt, Corse, and Chad, and reference sequences classified as *T. equi* found in Italy and Africa, with a nucleotide identity ranging from 99.2% to 100%. The two *T. haneyi*-like identified in cases of co-infection (448/2020 and 618/2020) were excluded from this analysis as the nucleotide sequences obtained were too short to be compared with other sequences.

**Figure 4:**
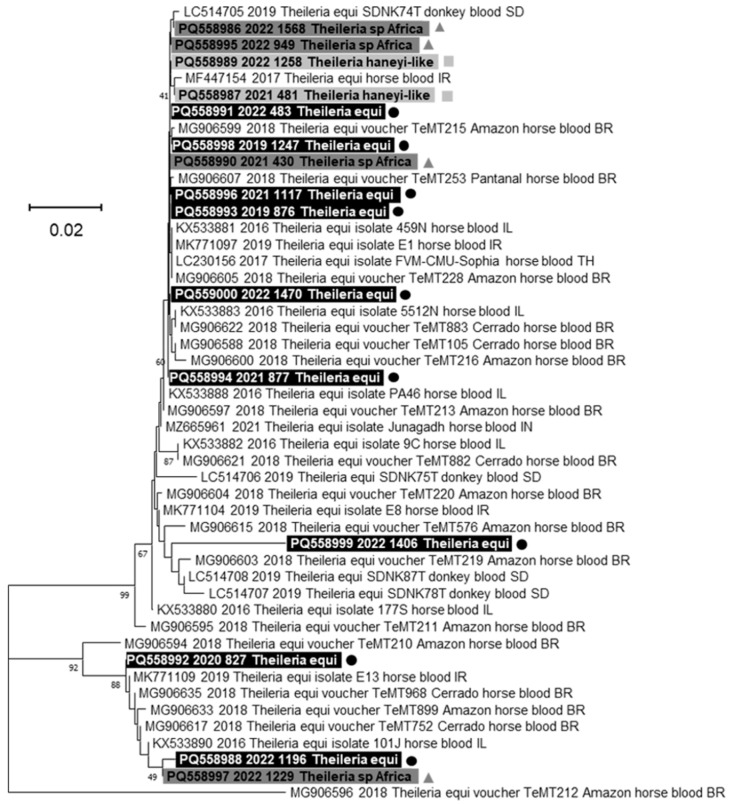
Phylogenetic tree constructed using the Neighbor-Joining method and the Kimura 2-parameter model implemented in MEGA 11 software version 11.0.11, based on the nucleotide alignment of 207 residues of the ema1 gene of *Theileria* spp. sequences obtained in this study with 33 reference sequences of *Theileria* spp. available in GenBank (https://www.ncbi.nlm.nih.gov/nucleotide/ (accessed on 15 November 2024)). Bootstrap values calculated from 1000 replicates are indicated on the respective branches. The reference sequences are labeled as follows: GenBank number, date of collection (or submission date), genus, species, host, sample matrix, country of origin (BR = Brazil, IL = Israel, IR = Iran, IT = Italy, SD = Sudan). Highlighted and marked sequences obtained in this study: with black ● for *T. equi*, with dark grey ▲ for *Theileria* sp. *Africa*, and with light grey ■ for *T. haneyi*-like, labeled as follows: GenBank Number (PQ558986-PQ559000), year of collection, sample ID, and 18S rRNA identification.

The phylogenetic tree constructed with a fragment of 209 pb of the *ema1* gene nucleotide sequences from *Theileria* spp. revealed that the sequences obtained in this study grouped in two different main clusters without any correlation with the genetic diversity that emerged from the 18SrRNA gene analysis (Figure 4).

## 4. Discussion

This study aimed to compare the diagnostic performance of different molecular assays, already validated in the literature for the detection of *Babesia* spp. and *Theileria* spp. DNA [20,21,22] in blood samples. The investigation was conducted on 63 horses exposed to risk factors for piroplasms infection or with clinical suspicion of EP collected in Italy from 2016 to 2022. Genetic characterization of the identified parasites through sequencing and phylogenetic analysis was performed.

The present study revealed that in the horse population examined, piroplasms belonging to both the *Babesia* and *Theileria* genera were circulating, with a total infection rate of 24/63 (38.1%). During the study period, there was a gradual increase in the number of animals tested, likely due to heightened awareness and sensitivity to these tick-borne pathogens, both among veterinary practitioners and animal owners. An increasing detection rate has been reported in Europe and some of the possible explanations can include climate change and the increased transport of horses, resulting in the spread of the competent tick vector [28]. The population included in this study showed significantly higher piroplasm positivity in females compared to male horses. This correlation between sex and positivity has been reported also in other studies [29,30]. However, another study on *B. caballi* infection states the opposite, finding more positives in male horses [31]. Furthermore, other authors reported no correlation between sex and positivity to piroplasms [32,33]. Given these conflicting results, it can be hypothesized that the sex of the horses does not directly influence their positivity to piroplasms. Instead, this appears to be a random finding, more closely associated with the purpose of the horse and environmental factors. In the literature, an increase in positivity with increasing age was reported, likely related to the persistence of infection, particularly for *Theileria* spp. infections [9,28]. In this study, the median age of positive horses observed (9 years and 6 months), being very close to the median age of the entire population included, may be influenced by the inclusion criteria adopted and potentially lead to non-representative and biased results.

From the comparison of the three different molecular assays used for diagnostic purposes (nested PCR, multiplex PCR, and real-time PCR), nPCR and qPCR were found to be the most effective methods for *Babesia* spp. DNA detection in blood samples, with sensitivity and specificity values of 100%. Differently, mPCR showed a low sensitivity value of 25% (95% CI = −0.174–0.674). This indicates that mPCR could fail to detect all true positive samples, leading to therapeutic issues. If piroplasmosis treatment is not promptly started, the animal condition could worsen, increasing the likelihood of death, especially in immunocompromised individuals [9]. The low sensitivity of this assay is probably linked to the fact that *B. caballi* infections often result in a low level of parasitemia in the host, with the parasite DNA levels remaining below its limit of detection [34]. On the contrary, the study found the best diagnostic performances in the detection of *Theileria* spp. DNA for mPCR, with 100% sensitivity and specificity values. For this piroplasm, the assay with the lowest performance was qPCR, which, being specific for *Babesia* spp., failed to detect any *Theileria* spp.-positive samples. Nested PCR was found to be a valid alternative assay, with a sensitivity value of 90.9% (95% CI = 81.8%–99.99%) and specificity of 100%. In the studied population, two (3.2%) horses were coinfected with *Babesia* and *Theileria* parasites. Such coinfections have been previously reported by different authors, with prevalence rates ranging from 2.5% in Egypt [25] to 20% in Cuba [35]. The simultaneous presence of two or more species of piroplasm can complicate the diagnosis and the treatment of the infected animal, leading to therapeutic failure or pathogen spread [36]. The present study identified one limit of nPCR: the assay was found to struggle in detecting co-infections between *B. caballi* and *Theileria* spp., probably because, in these cases, the parasite load of *B. caballi* was greater than that of *Theileria* spp. Indeed, the detection of *Theileria* spp. DNA in these samples was only possible through the use of specific primers for the genus *Theileria*.

Each assay used in this study for equine piroplasms DNA detection in horse blood samples showed advantages and disadvantages. Both nPCR and mPCR offered the advantage of detecting all equine piroplasms simultaneously, but each also had limitations. Specifically, nPCR showed excellent sensitivity for *B. caballi* DNA detection and good sensitivity for *Theileria* spp. DNA (with some difficulty in detecting co-infections), but it was time-consuming and costly, in addition to posing a higher risk of contamination and non-specific product amplification, which can complicate and lengthen the diagnostic process. Multiplex PCR was the most effective test for detecting *Theileria* spp. DNA, but it exhibited very low sensitivity for *B. caballi*. DNA detection. Finally, the qPCR evaluated in this study proved to be highly effective in *Babesia* spp. DNA detection, but it was unable to detect *Theileria* spp. DNA. Similarly, other assays comparative studies reported no single test currently available for sensitively and specifically detect and discriminate the different equine piroplasms species [7]. This highlights the need for further research to develop effective molecular assays to facilitate the diagnosis and subsequent treatment of infected horses.

Sequence analysis of the 18S rRNA gene revealed that most of the infections were caused by *Theileria* spp., with 20/63 (31.7%) horses tested positive, followed by *B. caballi* in 2/63 (3.2%) and two horses (2/63, 3.2%) with co-infections of *B. caballi* and *Theileria* spp. Horses positive for *Babesia caballi* DNA were infected with genetically identical piroplasms belonging to genotype B [14], indicating significant stability of this parasite over time and across different geographical regions. In contrast, horses positive for *Theileria* spp. DNA were infected with piroplasms from three distinct genetic groups: 9/63 (14.3%) with *T. equi*, 6/63 (9.5%) with *T. haneyi*-like, and 7/63 (11.1%) with *Theileria* sp. *Africa*. These findings enhance our understanding of piroplasm diversity in Italy, where few national studies have previously explored the prevalence of different *Theileria* species and genetic groups [30].

Preliminary studies on the 18S rRNA gene of equine piroplasms identified two distinct genotypes, including *Babesia* and *Theileria* species, respectively. These were first described in Spain by Nagore and colleagues [37] as *Babesia*-like and *Theileria*-like genotypes and confirmed by Kouam and colleagues [38] in Greece. Subsequently, Bhoora and colleagues [39] identified two genetic groups for *B. caballi*, designated as A and B, with the latter further subdivided into B1 and B2. For *Theileria equi*, three genetic groups were described and named as A, B, and C. To date, no further modifications have been proposed to the genetic grouping of *B. caballi*, except for the division of group A into A1 and A2 [14]. Nevertheless, significant genetic variability was reported for *B. caballi* subtype B, but no distinct subgroups were proposed [14]. On the contrary, modifications to the genetic grouping of *T. equi* have been proposed. Salim and colleagues [40] and Hall and colleagues [41] identified four genetic groups for *T. equi*, and subsequent studies by Qablan and colleagues [31] and Liu and colleagues [42] expanded this classification to five groups, designated A through E.

According to the latest classification proposed by Nehra and colleagues [15], the genetic groups identified in this study could be categorized as follows: *T. equi* as genotype A, *T. haneyi*-like as genotype B, and *Theileria* sp. Africa as genotype D. *T. haneyi* strains identified by Knowles and colleagues [2] could be categorized as genotype C. However, this classification is currently not universally accepted across all phylogenetic studies on these piroplasms, making it challenging to establish a consistent nomenclature due to discrepancies in the data and sequences available in the literature. Moreover, the proposal of *T. haneyi* as a new species suggests that many infections historically classified as *T. equi* may have been misidentified, complicating prevalence estimates and hindering the assessment of phylogenetic relationships [2].

Previous Italian studies conducted approximately 10 years ago did not differentiate between these two species and reported prevalence data exclusively for *B. caballi* and *T. equi*, findings that are consistent with the results of the present study. In the study by Ebani and colleagues [43], 26.87% of the animals tested were positive for *T. equi*, with no cases of *B. caballi* detected. In comparison, Zanet and colleagues [44] found 3.70% of horses positive for *B. caballi* and 13.33% for *T. equi*, while Sgorbini and colleagues [45] reported 1.9% positives for *B. caballi* and 32.2% for *T. equi*. Regarding *T. haneyi*, Elsawy and colleagues [25] in Egypt and Mshelia and colleagues [24] in Nigeria reported significant differences in molecular prevalence values, ranging from 20.3% of horses positive for *T. equi* compared to 53.1% positive for *T. haneyi* in the first study, to 13.3% positive for *T. equi* and 2.7% positive for *T. haneyi* in the second one. The discrepancy between these values highlights the need for further investigations to better understand the prevalence of this new parasite species.

Given these results, two PCR assays were carried out on genes specific for *T. equi* and *T. haneyi*, respectively, to try to complete the characterization of the piroplasms circulating in the equine population in Italy. A total of 16 out of 22 (72.7%) *Theileria*-positive samples tested positive with the *T. equi*-specific PCR targeting a fragment of the *ema1* gene, while none of the *Theileria*-positive samples tested positive with the *T. haneyi*-specific PCR targeting a fragment of the ORF of chromosome 1, probably because these strains belonged to a different species. Interestingly, while all the *T. equi* samples tested positive with the *ema1* gene PCR assay, only 2/6 (33.3%) of *T. haneyi-like* and 5/7 (71.4%) of *Theileria* sp. *Africa* samples showed specific amplicons. Similar results were also obtained by Manna and colleagues [13], who reported a correlation between the genotype identified through *18S rRNA* gene analysis and the positivity detected using molecular methods targeting the *ema1* gene. Furthermore, the phylogenetic analysis conducted in this study on the *ema1* gene revealed that there was no clustering associated with the genetic groups identified through the *18S rRNA* gene analysis. It is still unclear whether this correlation has any implications in terms of pathogenicity or transmissibility, as the few studies conducted so far reported conflicting results [13,46]. These results highlight challenges in the classification of these parasites, arising from their genetic variability, the scarcity or inaccuracy of data in the literature, and the limited sensitivity of available molecular methods. Furthermore, considering that *T. haneyi* lacks the *ema1* gene, which is often used as a diagnostic target for piroplasms, and considering the wide genetic diversity in the *18S rRNA* gene, the efficacy of the currently used molecular assays should be evaluated with caution. The analysis performed in this study revealed that, while there was no decrease in sensitivity with the tests used, distinguishing between *Theileria* species was not possible without sequencing and additional tests designed specifically for this genus.

From a therapeutic perspective, it is essential to identify the exact piroplasm species causing the infection in horses, as treatment protocols differ. Compared to *B. caballi*, *T. equi* has been found to be more resistant to treatment, requiring higher dosages and longer treatment times [6]. Moreover, recent studies have indicated that imidocarb dipropionate, typically used to treat *T. equi* infection, is not very effective against *T. haneyi* [47]. Given the treatment differences, it would be desirable to develop a molecular assay capable of detecting and distinguishing all the piroplasms species, even in cases of co-infection, in order to immediately initiate appropriate treatment without the need for sequencing.

The main limitation of this study was related to the included horse population, which, due to its retrospective nature, was biased by the previous selection of animals and a small sample size. The retrospective design may have introduced challenges, including the inclusion of animals that were not representative of the broader equine population. As a result, the observed molecular positivity rates may not accurately reflect those achievable in a larger or unselected population, with a possible overestimation of the infection rate due to a potential selection of cases with higher pathogen loads or evident clinical signs. Furthermore, the sensitivity and specificity of the diagnostic assays used in this study could not have been accurately estimated due to the limited sample size and the absence of a gold standard reference test to validate the accuracy of these measures. These limitations underline the importance of carrying out prospective studies with larger and randomly selected populations to better evaluate the epidemiological situation and the performance of the diagnostic assays used. Another limitation was associated with the short length of genomic fragments amplified and sequenced to perform phylogenetic analysis, in particular of the *ema1* gene, which may lead to errors in taxonomic definition.

## 5. Conclusions

In this study, the horse population examined had a higher number of infections caused by *Theileria* spp. than *Babesia* spp. and genetically different *Theileria* were detected in Italy, including *T. haneyi*-like and *Theileria* sp. Africa. Furthermore, the performance of different molecular assays used in this study to detect equine piroplasms DNA in blood samples was evaluated, and the best diagnostic approach to avoid false negative results and reduce diagnostic times was a combination of mPCR and qPCR. Further studies are necessary to better assess the prevalence and genetic diversity of piroplasms involved in equine piroplasmosis by expanding the tested population and analyzing longer genome fragments and different genes. It would be useful for both diagnosis and treatment purposes to develop a singular molecular assay capable of detecting and distinguishing all the *Babesia* and *Theileria* species with high levels of sensitivity and specificity. Additionally, since the available molecular assays showed suboptimal performances for both *Theileria* and *Babesia* DNA detection, the incorporation of blood smear analysis as a complementary diagnostic tool for morphological characterization of the parasites could be considered to improve the effectiveness of diagnosis at minimal additional cost.

## Figures and Tables

**Table 1:** Molecular assay used in this study.

Molecular Assay	Primers	Gene	Fragment Length	Reference
nPCR for *Babesia* and *Theileria* species detection	PCR 1	18S rRNA	930 bp	[20]
	BTF1 = GGC TCA TTA CAA CAG TTA TAG BTR1 = CCC AAA GAC TTT GAT TTC TCT C			
	PCR 2		800 bp	
	BTF2 = CCG TGC TAA TTG TAG GGC TAA TAC BTR2 = GGA CTA CGA CGG TAT CTG ATC G			
				
mPCR for *B. caballi* and *T. equi* detection	Bec-UF2 = TCG AAG ACG ATC AGA TAC CGT CG Cab-R = CTC GTT CAT GAT TTA GAA TTG CT Equi-R = TGC CTT AAA CTT CTT TGC GAT	18S rRNA	540 bp *B. caballi* 450 bp *T. equi*	[21]
				
qPCR for *Babesia* spp. detection	B-lsu-F = ACC TGT CAA RTT CCT TCA CTA AMT T B-lsu-R2 = TCT TAA CCC AAC TCA CGT ACC A	*lsu*	150 bp	[22]
				
PCR *T. equi*-specific	PCR 1—External primers Th_ema1_for = GAG GAG GAG AAA CCC AAG Th_ema1_rev = GCC ATC GCC CTT GTA GAG PCR 2—Internal primers Th_ema1_for = TCA AGG ACA ACA AGC CAT AC Th_ema1_rev = TTG CCT GGA GCC TTG AAG	*ema1*	229 bp	[23]
				
PCR *T. haneyi*-specific	PCR 1—External primers Th_ORF_ch1_for = CCA TAC AAC CCA CTA GAG Th_ORF_ch1_rev = CTG TCA TTT GGG TTT GAT AG PCR 2—Internal primers Th_ORF_ch1_for = GAC AAC AGA GAG GTG ATT Th_ORF_ch1_rev = CGT TGA ATG TAA TGG GAA C	*ORF chromosome 1*	238 bp	[2]

bp: basepair; *ema1* = equi merozoite antigen-1; lsu = large subunit ribosomal DNA; mPCR = multiplex PCR; nPCR = nested PCR; qPCR = real-time PCR.

**Table 2:** Results of molecular screening.

	18S rRNA Gene				lsu Gene	*ema1* Gene	*ORF Chromosome 1* Gene
Horses	nPCR		mPCR		q PCR	PCR	PCR
	*Babesia* spp.	*Theileria* spp.	*B. caballi*	*T. equi*	*Babesia* spp.	*T. equi*	*T. haneyi*
1630/2022	0	1	0	1	0	0	0
1470/2022	0	1	0	1	0	1	0
1406/2022	0	1	0	1	0	1	0
1258/2022	0	1	0	1	0	1	0
1229/2022	0	1	0	1	0	1	0
1196/2022	0	1	0	1	0	1	0
949/2022	0	1	0	1	0	1	0
483/2022	0	1	0	1	0	1	0
482/2022	0	1	0	1	0	0	0
1568/2021	0	1	0	1	0	1	0
1117/2021	0	1	0	1	0	1	0
877/2021	0	1	0	1	0	1	0
481/2021	0	1	0	1	0	1	0
430/2021	0	1	0	1	0	1	0
827/2020	0	1	0	1	0	1	0
802/2020	1	0	0	0	1	NT	NT
782/2020	0	1	0	1	0	0	0
618/2020	1	0	0	1	1	0	0
523/2020	1	0	1	0	1	NT	NT
448/2020	1	0	0	1	1	0	0
1247/2019	0	1	0	1	0	1	0
876/2019	0	1	0	1	0	1	0
346/2019	0	1	0	1	0	0	0
539/2017	0	1	0	1	0	1	0
Tot.	4	20	1	22	4	16	0

0 = negative; 1 = positive; mPCR = multiplex PCR; nPCR = nested PCR; NT = not tested; PCR: end-point PCR; qPCR = real-time PCR.

**Table 3:** Sensitivity and specificity values for each molecular assay used for *Babesia* spp. and *Theileria* spp. DNA detection.

		*Babesia* spp.	*Theileria* spp.
nPCR	rSe	100%	90.9% (0.79–1.029)
	rSp	100%	100%
mPCR	rSe	25% (−0.174–0.674)	100%
	rSp	100%	100%
qPCR	rSe	100%	0%
	rSp	100%	100%

mPCR = multiplex PCR; nPCR = nested PCR; PCR = end-point PCR; qPCR = real-time PCR; rSe = relative sensibility; rSp = relative specificity. In brackets, confidence intervals.

**Table 4:** Results of the 18S rRNA gene sequence analysis of the identified piroplasms.

Sample	Sequencing Result
1630/2022	*Theileria* sp. Africa
1470/2022	*T. equi*
1406/2022	*T. equi*
1258/2022	*T. haneyi*-like
1229/2022	*Theileria* sp. Africa
1196/2022	*T. equi*
949/2022	*Theileria* sp. Africa
483/2022	*T. equi*
482/2022	*Theileria* sp. Africa
1568/2021	*Theileria* sp. Africa
1117/2021	*T. equi*
877/2021	*T. equi*
481/2021	*T. haneyi*-like
430/2021	*Theileria* sp. Africa
827/2020	*T. equi*
802/2020	*B. caballi*
782/2020	*T. haneyi*-like
618/2020	*B. caballi + T. haneyi*-like
523/2020	*B. caballi*
448/2020	*B. caballi + T. haneyi*-like
1247/2019	*T. equi*
876/2019	*T. equi*
346/2019	*T. haneyi*-like
539/2017	*Theileria* sp. Africa

## Data Availability

All data generated or analyzed during this study are included in this published article. The original data presented in the study are openly available in AMSActa UNIBO at [https://doi.org/10.6092/unibo/amsacta/7989 (accessed on 13 November 2024)]. The nucleotide sequences generated and analyzed during the current study are available in the International Nucleotide Sequence Database Collaboration repository (INSDC, http://www.insdc.org/ (accessed on 25 November 2024)) with the IDs: PQ555641-PQ555666, PQ558986-PQ559000.

## References

[1] Padalino B., Rosanowski S.M., Di Bella C., Lacinio R., Rubino G.T. (2019). Piroplasmosis in Italian Standardbred horses: 15 years of surveillance data. JEVS.

[2] Knowles D.P., Kappmeyer L.S., Haney D., Herndon D.R., Fry L.M., Munro J.B., Sears K., Ueti M.W., Wise L.N., Silva M. (2018). Discovery of a novel species, *Theileria haneyi* n. sp., infective to equids, highlights exceptional genomic diversity within the genus *Theileria*: Implications for apicomplexan parasite surveillance. Int. J. Parasitol..

[3] Schnittger L., Rodriguez A.E., Florin-Christensen M., Morrison D.A. (2012). Babesia: A world emerging. Infect. Gen. Evol..

[4] Wise L.N., Kappmeyer L.S., Mealey R.H., Knowles D.P. (2013). Review of equine piroplasmosis. J. Vet. Intern. Med..

[5] Nadal C., Bonnet S.I., Marsot M. (2021). Eco-epidemiology of equine piroplasmosis and its associated tick vectors in Europe: A systematic literature review and a meta-analysis of prevalence. Transbound. Emerg. Dis..

[6] Tirosh-Levy S., Gottlieb Y., Fry L.M., Knowles D.P., Steinman A. (2020). Twenty years of equine piroplasmosis research: Global distribution, molecular diagnosis, and phylogeny. Pathogens.

[7] Nardini R., Bartolomé Del Pino L.E., Cersini A., Manna G., Viola M.R., Antognetti V., Autorino G.L., Scicluna M.T. (2021). Comparison of PCR-based methods for the detection of *Babesia caballi* and *Theileria equi* in field samples collected in Central Italy. Parasitol. Res..

[8] Nardini R., Cersini A., Bartolomé Del Pino L.E., Manna G., Scarpulla M., Di Egidio A., Giordani R., Antognetti V., Veneziano V., Scicluna M.T. (2022). Comparison of direct and indirect methods to maximise the detection of *Babesia caballi* and *Theileria equi* infections in Central Southern Italy. Ticks Tick-Borne Dis..

[9] Onyiche T.E., Suganuma K., Igarashi I., Yokoyama N., Xuan X., Thekisoe O. (2019). A review on equine piroplasmosis: Epidemiology, vector ecology, risk factors, host immunity, diagnosis and control. Int. J. Environ. Res. Public Health.

[10] Rothschild C.M. (2013). Equine piroplasmosis. JEVS.

[11] Ali S., Sugimoto C., Onuma M. (2001). Equine piroplasmosis. JEVS.

[12] Jaffer O., Abdishakur F., Hakimuddin F., Riya A., Wernery U., Schuster R.K. (2010). A comparative study of serological tests and PCR for the diagnosis of equine piroplasmosis. Parasitol. Res..

[13] Manna G., Cersini A., Nardini R., Bartolomé Del Pino L.E., Antognetti V., Zini M., Conti R., Lorenzetti R., Veneziano V., Autorino G.L. (2018). Genetic diversity of *Theileria equi* and *Babesia caballi* infecting horses of Central-Southern Italy and preliminary results of its correlation with clinical and serological status. Ticks Tick-Borne Dis..

[14] Nehra A., Kumari A., Moudgil A., Vohra S. (2021). Phylogenetic analysis, genetic diversity and geographical distribution of *Babesia caballi* based on 18S rRNA gene. Ticks Tick-Borne Dis..

[15] Nehra A.K., Kumari A., Moudgil A.D., Vohra S. (2024). Revisiting the genotypes of *Theileria equi* based on the V4 hypervariable region of the 18S rRNA gene. Front. Vet. Sci..

[16] Kumar B., Maharana B.R., Thakre B., Brahmbhatt N.N., Joseph J.P. (2022). 18S rRNA gene-based piroplasmid PCR: An assay for rapid and precise molecular screening of Theileria and Babesia species in animals. Acta Parasitol..

[17] Ebrahimi M., Adinehbeigi K., Hamidinejat H., Tabandeh M.R. (2018). Molecular characterization of *Theileria equi* infection in horse populations belonging to West Azerbaijan, Iran: Insights into the importance of Equine Merozoite Antigen (EMA)-1 in its diagnosis. Ann. Parasitol..

[18] Pavan V., Antolini G., Barbiero R., Berni N., Brunier F., Cacciamani C., Cagnati A., Cazzuli O., Cicogna A., De Luigi C. (2018). High resolution climate precipitation analysis for north-central Italy, 1961–2015. Clim. Dyn..

[19] Zobba R., Ardu M., Niccolini S., Chessa B., Manna L., Cocco R., Parpaglia M.L.P. (2008). Clinical and laboratory findings in equine piroplasmosis. JEVS.

[20] Jefferies R., Ryan U.M., Irwin P.J. (2007). PCR-RFLP for the detection and differentiation of the canine piroplasm species and its use with filter paper-based technologies. Vet. Parasitol..

[21] Alhassan A., Pumidonming W., Okamura M., Hirata H., Battsetseg B., Fujisaki K., Yokoyama N., Igarashi I. (2005). Development of a single-round and multiplex PCR method for the simultaneous detection of *Babesia caballi* and Babesia equi in horse blood. Vet. Parasitol..

[22] Qurollo B.A., Archer N.R., Schreeg M.E., Marr H.S., Birkenheuer A.J., Haney K.N., Thomas B.S., Breitschwerdt E.B. (2017). Improved molecular detection of Babesia infections in animals using a novel quantitative real-time PCR diagnostic assay targeting mitochondrial DNA. Parasit. Vectors.

[23] Baptista C., Lopes M.S., Tavares A.C., Rojer H., Kappmeyer L., Mendonça D., da Câmara Machado A. (2013). Diagnosis of *Theileria equi* infections in horses in the Azores using cELISA and nested PCR. Ticks Tick-Borne Dis..

[24] Mshelia P.W., Kappmeyer L., Johnson W.C., Kudi C.A., Oluyinka O.O., Balogun E.O., Richard E.E., Onoja E., Sears K.P., Ueti M.W. (2020). Molecular detection of Theileria species and *Babesia caballi* from horses in Nigeria. Parasitol. Res..

[25] Elsawy B.S., Nassar A.M., Alzan H.F., Bhoora R.V., Ozubek S., Mahmoud M.S., Kandil O.M., Mahdy O.A. (2021). Rapid detection of equine piroplasms using multiplex PCR and first genetic characterization of *Theileria haneyi* in Egypt. Pathogens.

[26] Tamura K., Stecher G., Kumar S. (2021). MEGA11: Molecular evolutionary genetics analysis version 11. Mol. Biol. Evol..

[27] Newcombe R.G. (1998). Two-sided confidence intervals for the single proportion: Comparison of seven methods. Stat. Med..

[28] Axt C.W., Springer A., Strube C., Jung C., Naucke T.J., Müller E., Schäfer I. (2024). Molecular and Serological Detection of Vector-Borne Pathogens Responsible for Equine Piroplasmosis in Europe between 2008 and 2021. Microorganisms.

[29] Rüegg S.R., Torgerson P., Deplazes P., Mathis A. (2007). Age-dependent dynamics of *Theileria equi* and *Babesia caballi* infections in southwest Mongolia based on IFAT and/or PCR prevalence data from domestic horses and ticks. Parasitology.

[30] Moretti A., Mangili V., Salvatori R., Maresca C., Scoccia E., Torina A., Moretta I., Gabrielli S., Tampieri M.P., Pietrobelli M. (2010). Prevalence and diagnosis of Babesia and Theileria infections in horses in Italy: A preliminary study. Vet. J..

[31] Qablan M.A., Oborník M., Petrželková K.J., Sloboda M., Shudiefat M.F., Hořín P., Lukeš J., Modrý D. (2013). Infections by *Babesia caballi* and *Theileria equi* in Jordanian equids: Epidemiology and genetic diversity. Parasitology.

[32] Grandi G., Molinari G., Tittarelli M., Sassera D., Kramer L.H. (2011). Prevalence of *Theileria equi* and *Babesia caballi* infection in horses from northern Italy. Vector Borne Zoonotic Dis..

[33] Bahrami S., Ghadrdan A.R., Mirabdollahi S.M., Fayed M.R. (2014). Diagnosis of subclinical equine theileriosis in center of Iran using parasitological and molecular methods. Trop. Biomed..

[34] Del Pino L.E.B., Meana A., Zini M., Cersini A. (2023). Evidence of transplacental transmission of equine piroplasms *Theileria equi* and *Babesia caballi* in an Italian breed mare. Folia Parasitol..

[35] Díaz-Sánchez A., Pires M., Estrada C., Cañizares E., Domínguez S., Cabezas-Cruz A., Rivero E., Fonseca A., Massard C., Corona-González B. (2018). First molecular evidence of *Babesia caballi* and *Theileria equi* infections in horses in Cuba. Parasitol. Res..

[36] Idoko I., Edeh R., Adamu A., Machunga-Mambula S., Okubanjo O., Balogun E., Adamu S., Johnson W., Kappmeyer L., Mousel M. (2021). Molecular and Serological Detection of Piroplasms in Horses from Nigeria. Pathogens.

[37] Nagore D., García-Sanmartín J., García-Pérez A.L., Juste R.A., Hurtado A. (2004). Detection and identification of equine Theileria and Babesia species by reverse line blotting: Epidemiological survey and phylogenetic analysis. Vet. Parasitol..

[38] Kouam M.K., Kantzoura V., Masuoka P.M., Gajadhar A.A., Theodoropoulos G. (2010). Genetic diversity of equine piroplasms in Greece with a note on speciation within *Theileria* genotypes (*T. equi* and *T. equi*-like). Infect. Genet. Evol..

[39] Bhoora R., Franssen L., Oosthuizen M.C., Guthrie A.J., Zweygarth E., Penzhorn B.L., Jongejan F., Collins N.E. (2009). Sequence heterogeneity in the 18S rRNA gene within *Theileria equi* and *Babesia caballi* from horses in South Africa. Vet. Parasitol..

[40] Salim B., Bakheit M.A., Kamau J., Nakamura I., Sugimoto C. (2010). Nucleotide sequence heterogeneity in the small subunit ribosomal RNA gene within *Theileria equi* from horses in Sudan. Parasitol. Res..

[41] Hall C.M., Busch J.D., Scoles G.A., Palma-Cagle K.A., Ueti M.W., Kappmeyer L.S., Wagner D.M. (2013). Genetic characterization of *Theileria equi* infecting horses in North America: Evidence for a limited source of U.S. introductions. Parasit. Vectors.

[42] Liu Q., Meli M.L., Zhang Y., Meili T., Stirn M., Riond B., Weibel B., Hofmann-Lehmann R. (2016). Sequence heterogeneity in the 18S rRNA gene in *Theileria equi* from horses presented in Switzerland. Vet. Parasitol..

[43] Ebani V.V., Nardoni S., Bertelloni F., Rocchigiani G., Mancianti F. (2015). Tick-borne infections in horses from Tuscany, Italy. JEVS.

[44] Zanet S., Bassano M., Trisciuoglio A., Taricco I., Ferroglio E. (2017). Horses infected by Piroplasms different from *Babesia caballi* and *Theileria equi*: Species identification and risk factors analysis in Italy. Vet. Parasitol..

[45] Sgorbini M., Bonelli F., Nardoni S., Rocchigiani G., Corazza M., Mancianti F. (2015). Seroprevalence and molecular analysis of *Babesia caballi* and *Theileria equi* in horses from central Italy during a 10-year period. JEVS.

[46] Bhoora R., Quan M., Matjila P.T., Zweygarth E., Guthrie A.J., Collins N.E. (2010). Sequence heterogeneity in the equi merozoite antigen gene (*ema*-1) of *Theileria equi* and development of an *ema*-1-specific TaqMan MGB assay for the detection of *T. equi*. Vet. Parasitol..

[47] Sears K., Knowles D., Dinkel K., Mshelia P.W., Onzere C., Silva M., Fry L. (2020). Imidocarb Dipropionate Lacks Efficacy against *Theileria haneyi* and Fails to Consistently Clear *Theileria equi* in Horses Co-Infected with *T. haneyi*. Pathogens.

